# Bone-Preserving Robotic Conversion of Medial UKA to TKA: A Step-by-Step Technique

**DOI:** 10.3390/jcm14217887

**Published:** 2025-11-06

**Authors:** Jaad Mahlouly, Alexander Antoniadis, Thibaut Royon, Julien Wegrzyn

**Affiliations:** Department of Orthopedic Surgery and Trauma, Lausanne University Hospital and University of Lausanne, 1005 Lausanne, Switzerlandjulien.wegrzyn@chuv.ch (J.W.)

**Keywords:** robotic-assisted TKA, UKA revision, functional alignment, CT-based planning, bone grafting, primary cementless components

## Abstract

Converting a medial unicompartmental knee arthroplasty (UKA) to total knee arthroplasty (TKA) presents challenges in bone preservation, alignment, and soft-tissue balance. Robotic assistance enables three-dimensional CT-based planning, precise bone preparation, and real-time ligament balance assessment, thereby supporting a bone-preserving approach. We describe a stepwise workflow using the Mako system (Stryker) to convert a failed medial UKA to a condylar-stabilizing (CS) Triathlon TKA within a functional alignment framework. Pre-explantation registration on the in situ components maintains accuracy despite potential metallic artifacts. The polyethylene insert is briefly removed solely to access reference surfaces, then registration landmarks are acquired directly on the metal components along the marked central sagittal axis. The insert is reinserted to enable dynamic intraoperative balance evaluation. After component removal, a small medial tibial or femoral defect can be filled with autologous cancellous bone graft from the resected bone surfaces. Definitive cementless components are implanted without stems or augments, and the patella is resurfaced. This technique provides a reproducible robotic workflow for UKA-to-TKA conversion in selected cases with preserved bone stock and stable soft-tissue balance and facilitates accurate and reproducible conversion with optimal bone preservation.

## 1. Introduction

Conversion of a failed medial unicompartmental knee arthroplasty (UKA) to total knee arthroplasty (TKA) presents several technical challenges, such as implant removal, management of bone defects, restoration of alignment, and soft-tissue balancing. These cases often necessitate revision-type components with stems or augments, even in the absence of loosening or infection [1,2,3,4].

Advances in robotic-assisted TKA, including CT-based three-dimensional planning, real-time balance assessment, and precise bone preparation, offer new opportunities for a bone-preserving strategy in this setting [5,6,7]. When applied within a functional alignment framework, 3D planning enables resections on healthy bone, optimizing fixation conditions and potentially allowing the use of cementless primary implants without augments or stems—an outcome less likely with conventional mechanical alignment [8,9].

The present technical note describes a step-by-step workflow for robotic-assisted conversion of medial UKA to TKA using standard condylar-stabilized components without stems or augments. The approach was inspired by the work of Marchand’s group and refined through institutional experience in high-volume robotic-assisted TKA, leading us to adopt a functional alignment philosophy based on native bone morphology and physiological ligament balance [8]. As highlighted by Shatrov et al., this alignment strategy may reduce the need for augments or ligament releases in appropriately selected cases [9]. Nevertheless, its successful application requires a thorough understanding of robotic workflow and an appreciation of the learning curve associated with UKA-to-TKA conversion [6,10,11]. To our knowledge, no previous technical note has detailed a robotic-assisted UKA-to-TKA conversion workflow that combines pre-explantation registration, functional alignment, and exclusive use of cementless primary components without stems or augments.

## 2. Surgical Technique

This protocol was developed to enable a bone-preserving UKA-to-TKA conversion workflow using advanced robotic assistance. The strategy emphasizes functional alignment, precise 3D preoperative planning, and dynamic intraoperative adjustment based on real-time functional alignment and soft-tissue assessment, as described by recent reference techniques [8,9].

### 2.1. Pre-Operative Planning

Pre-operative planning begins with the acquisition of a standard Hip-Knee-Ankle protocol CT scan, imported into the Mako robotic software (Mako 3.1—Stryker, Mahwah, NJ, USA). No dedicated metal–artifact–reduction protocol is applied during CT acquisition. Artifact mitigation is performed during image processing after acquisition at the reconstruction console: we generate a standard bone-kernel series and a MAR-processed series at the knee, and any residual artifacts from the in situ UKA are taken into account during registration planning. Three-dimensional reconstruction allows precise assessment of bone stock, implant position, and soft-tissue envelope. The surgical objective is to restore functional alignment based on native bone morphology and physiological ligament tension. The plan is refined to maximize bone preservation and minimize the need for stems or augments. Target parameters include restoring the hip–knee–ankle angle within 180° ± 5°, maintaining joint line height within ±2 mm, and achieving 1–2 mm of controlled laxity with symmetric medial and lateral gaps (side-to-side difference ≤ 1 mm) in terminal extension and 90° of flexion while avoiding mid-flexion instability.

### 2.2. Patient Positioning and Approach

The patient is positioned supine, without a tourniquet. A medial parapatellar approach is used, re-entering the prior incision. The patella is retracted laterally without eversion. Ensure stable fixation of the limb to allow smooth robotic arm tracking and full range of motion during intraoperative assessment.

### 2.3. Pins Placement and Landmark Registration

**Figure 1:**
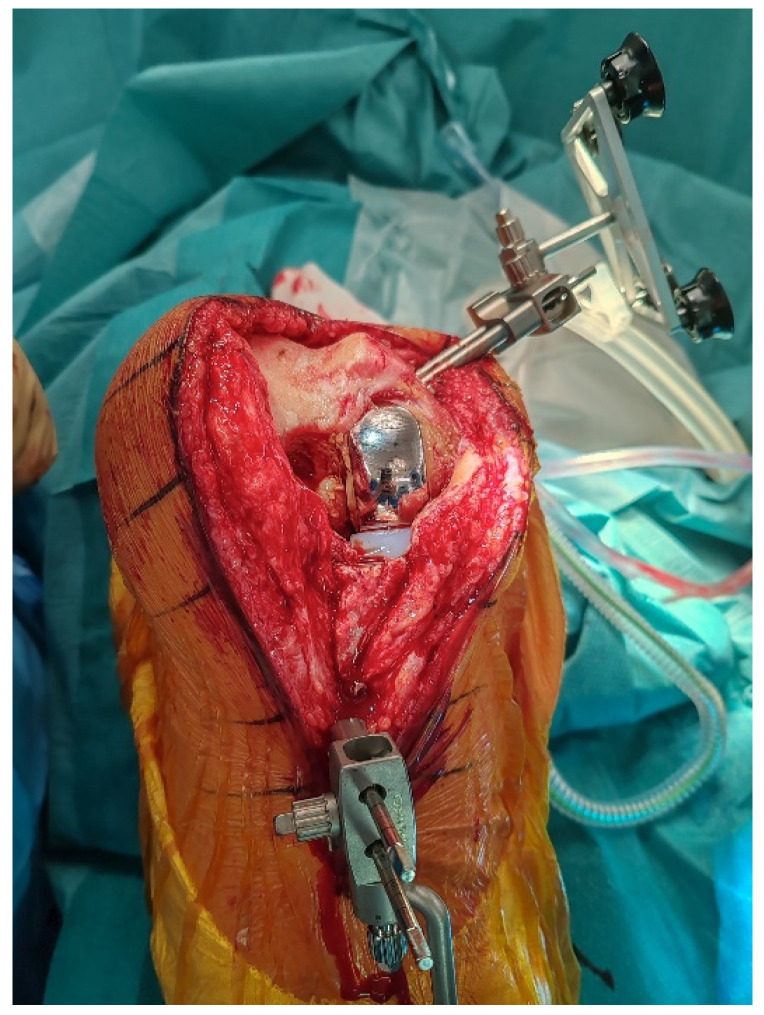
Intraoperative view after medial parapatellar approach reusing the previous incision and arthrotomy, showing the medial unicompartmental knee arthroplasty (UKA) in situ with array pins in place, prior to registration and component removal.

**Figure 2:**
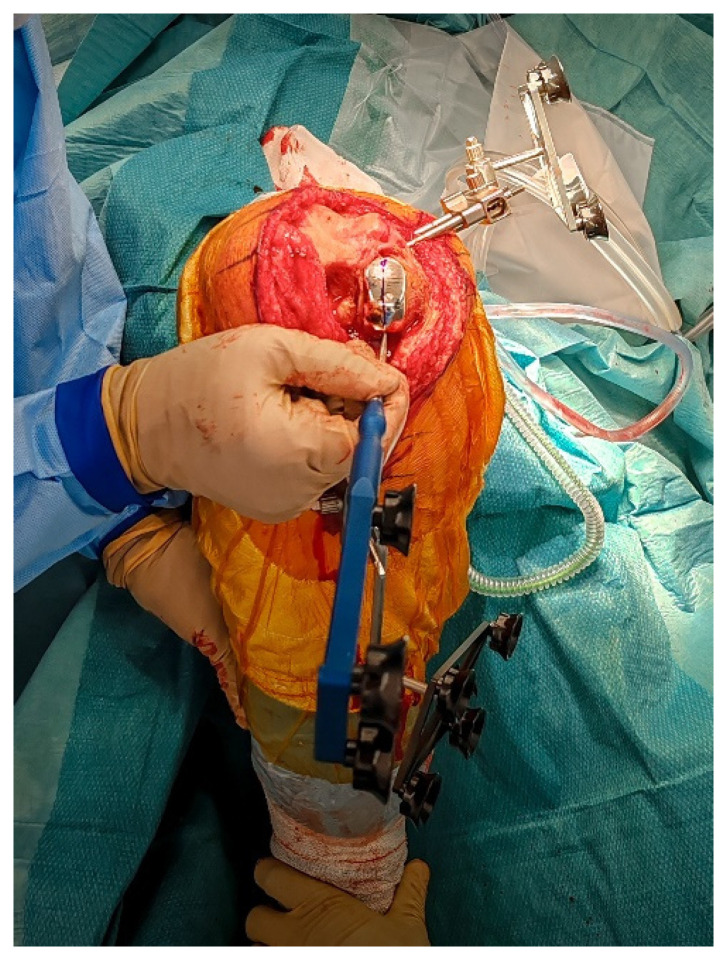
The polyethylene insert is removed to access reference surfaces. Registration landmarks are then acquired directly on the in situ metal components along the marked central sagittal axis.

Array pins for the robotic system are inserted according to protocol (4 mm femur, 3 mm tibia) through the same medial parapatellar incision to minimize soft-tissue trauma (Figure 1). Before explantation, registration is performed using the central sagittal axis of the in situ UKA components to limit the impact of CT metal artifacts (Figure 2). On the femoral component, this axis is marked with a dermographic pen. The polyethylene insert is removed solely to expose reference surfaces. Registration landmarks are acquired directly on the in situ metal components, along the pre-marked central sagittal axis (used as a surrogate of the native medial condyle) to improve mapping accuracy. The polyethylene insert is reinserted immediately afterward to allow dynamic balance assessment. Pin stability is verified prior to proceeding, because any motion can compromise tracking accuracy throughout the procedure. Registration accuracy is confirmed with checkpoint validation. If any control checkpoint exceeds 0.5 mm, landmarks and checkpoints are repeated until all residuals meet tolerance. Osteophytes are left in place during registration and removed only afterward to avoid altering reference points.

### 2.4. Intraoperative Functional Alignment and Ligament Balance Assessment

**Figure 3:**
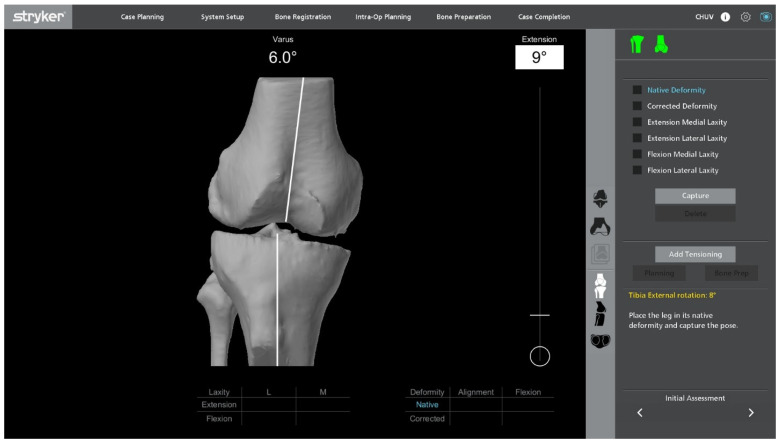
Intraoperative ligament balance assessment after registration. The polyethylene insert is reinserted prior to implant removal to evaluate dynamic ligament balancing through flexion and extension.

With the UKA components in place, an initial intraoperative functional alignment assessment is performed (Figure 3). The polyethylene insert is reinserted to enable a dynamic evaluation of ligament balance in both flexion and extension, providing essential data for potential plan adjustments. After registration and verification of reference points, this assessment is repeated through the full range of motion, allowing real-time adaptation to refine the 3D plan.

Varus–valgus stress tests are applied at multiple flexion angles to assess ligament balance. The target is 1–2 mm of controlled laxity with symmetric medial and lateral gaps in extension and at 90° of flexion. The medial–lateral side-to-side difference should be ≤1 mm to maintain optimal soft-tissue tension and avoid mid-flexion instability.

Within a functional-alignment strategy, balance is restored by adjusting implant positioning and resection targets rather than by performing ligament or soft-tissue releases. This bone-preserving functional-alignment strategy maintains the native joint line and creates optimal conditions for accurate, conservative implantation.

### 2.5. Implant Removal

**Figure 4:**
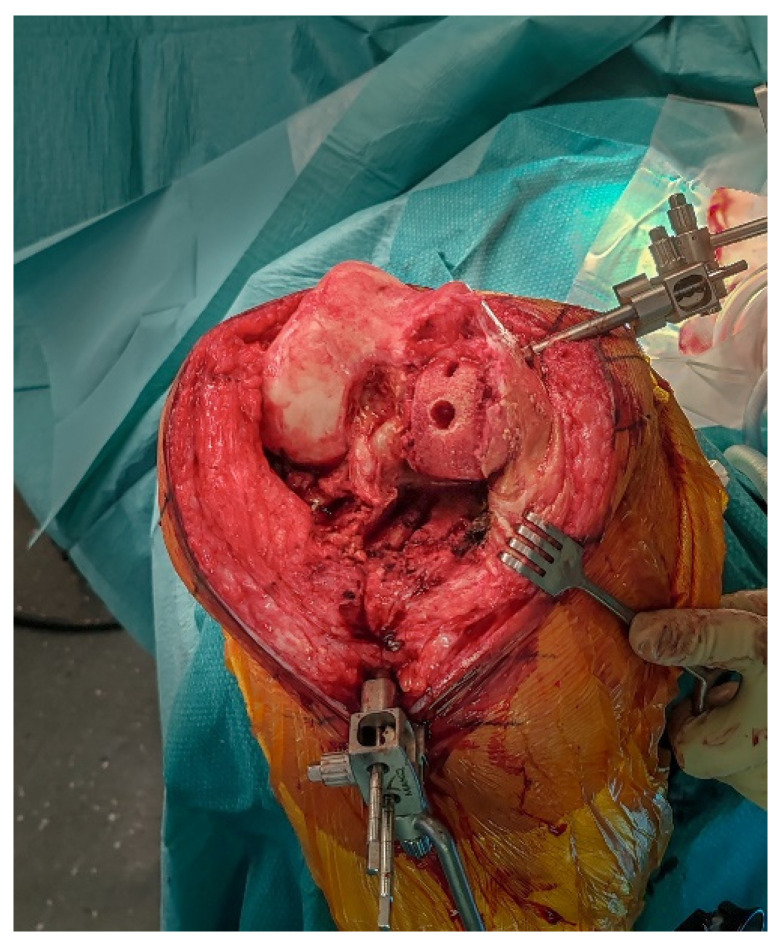
Intraoperative view after removal of the medial UKA components, showing bone-sparing removal with preserved host bone.

The femoral and tibial components are removed using thin osteotomes and an oscillating saw, preserving as much bone as possible (Figure 4). Careful sequential release of the bone–cement interface, combined with minimal lever forces, helps reduce the risk of iatrogenic bone defects.

### 2.6. Robotic Planning and Bone Resection

**Figure 5:**
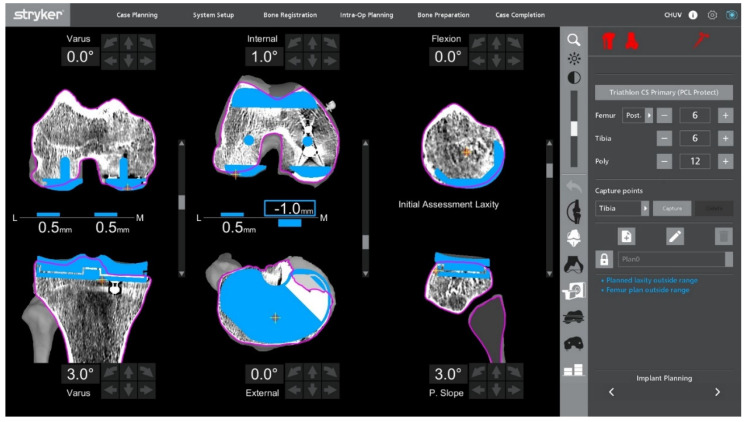
Final adjusted implant positioning and robotic resection plan after gap assessment, intentionally showing sub-millimetric residual gaps at the no-stress stage. Planning aims optimize ligament balance while preserving bone.

After confirming ligament balance, the preoperative plan is adjusted if necessary to optimize bone preservation and soft-tissue tension. Robotic-guided resections are performed according to the final plan (Figure 5). The intraoperative screen may display a plan with submillimetric residual gaps, which is an intended target and acceptable without stress testing at this stage. At 90° of flexion, a small sub-millimetric lateral laxity is acceptable and often desirable to permit a physiological medial-pivot pattern while maintaining coronal stability.

### 2.7. Bone Defect Management

**Figure 6:**
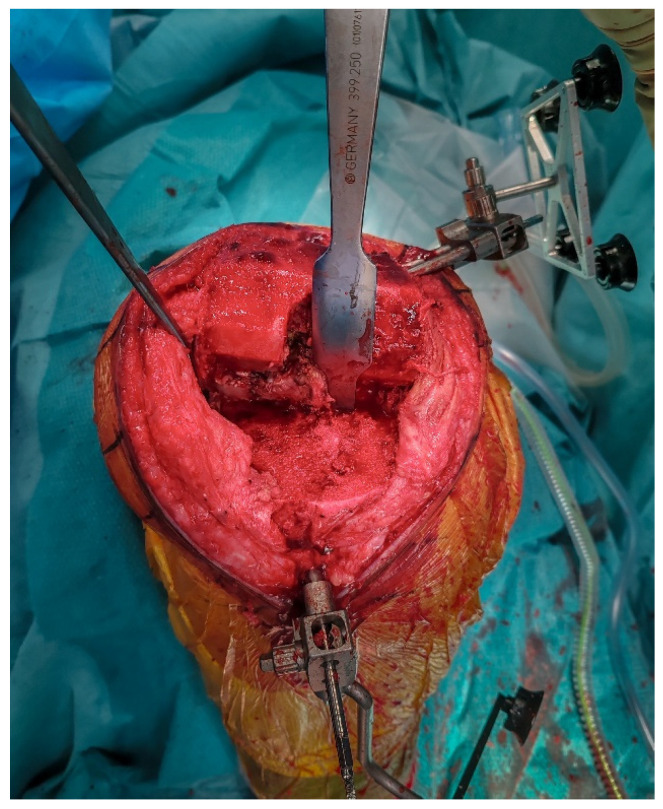
Autologous cancellous bone graft harvested from femoral resections is impacted into the medial tibial defect left by the UKA keel.

Following explantation, any residual metaphyseal defect, most commonly located at the site of the former UKA tibial keel, is managed using autologous cancellous bone graft harvested from the femoral resections. The graft is then impacted with a tamp and mallet to recreate a stable and supportive bed for the tibial baseplate (Figure 6).

### 2.8. Final Implantation

**Figure 7:**
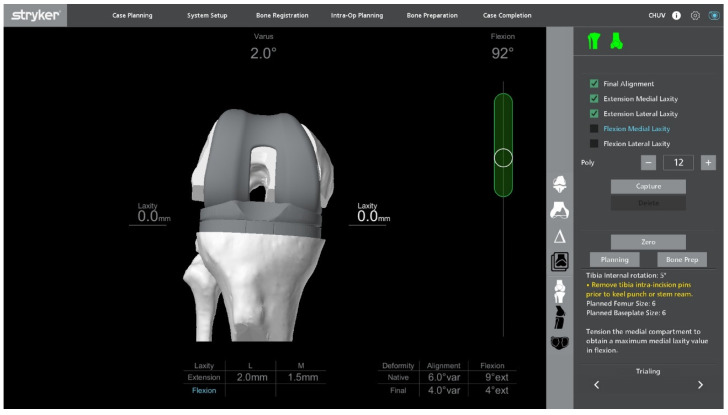
Ligament balancing testing with trial components in place, confirming stable flexion–extension gaps prior to final implantation.

**Figure 8:**
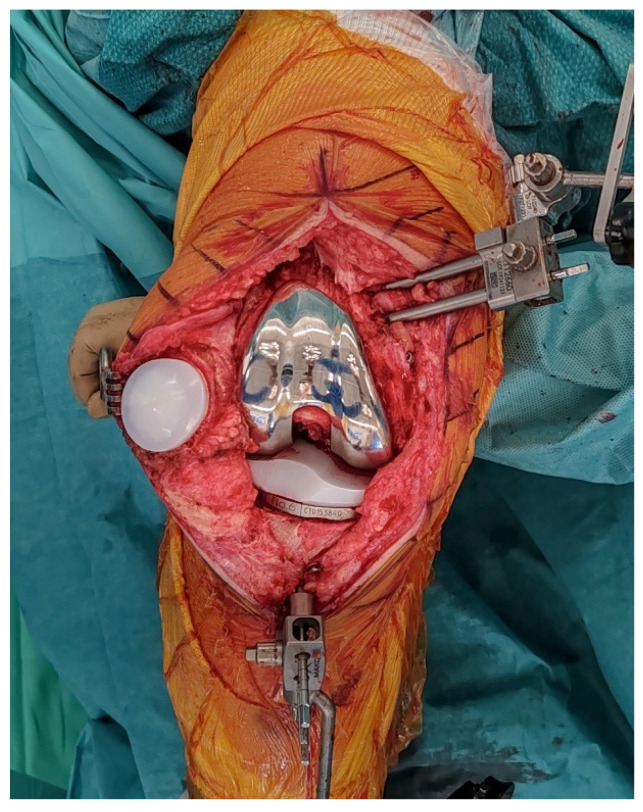
Final implantation of CS TKA with patellar resurfacing.

Trial components are inserted to confirm flexion–extension gap balance and overall stability (Figure 7). At this stage, balance is assessed both without stress and under standardized varus-valgus stress in terminal extension and at 90° of flexion. Once satisfactory alignment and soft-tissue tension are achieved, definitive cementless condylar-stabilizing components are implanted (Figure 8). We favor CS inserts to obtain coronal stability without ligament releases and without a cam-post mechanism, consistent with bone preservation. Cruciate-retaining implants are used only when the posterior cruciate ligament is clearly competent, and posterior-stabilized (or revision) constructs are selected when anteroposterior stability cannot be ensured with CS inserts or when ligament competence or bone stock are insufficient. Stems, augments, or ligament releases are typically not required. The patella is systematically resurfaced to decrease the risk of future anterior knee pain and revision surgery. Ensure that the final implant position matches the 3D planning and that all the implant press-fit interfaces are fully seated before closure.

### 2.9. Irrigation and Closure

The joint is irrigated with saline. We check for hemostasis. The capsule is closed in a watertight fashion, followed by the subcutaneous tissues and skin, using staples. A hydrocolloid dressing is applied for wound protection.

**Figure 9:**
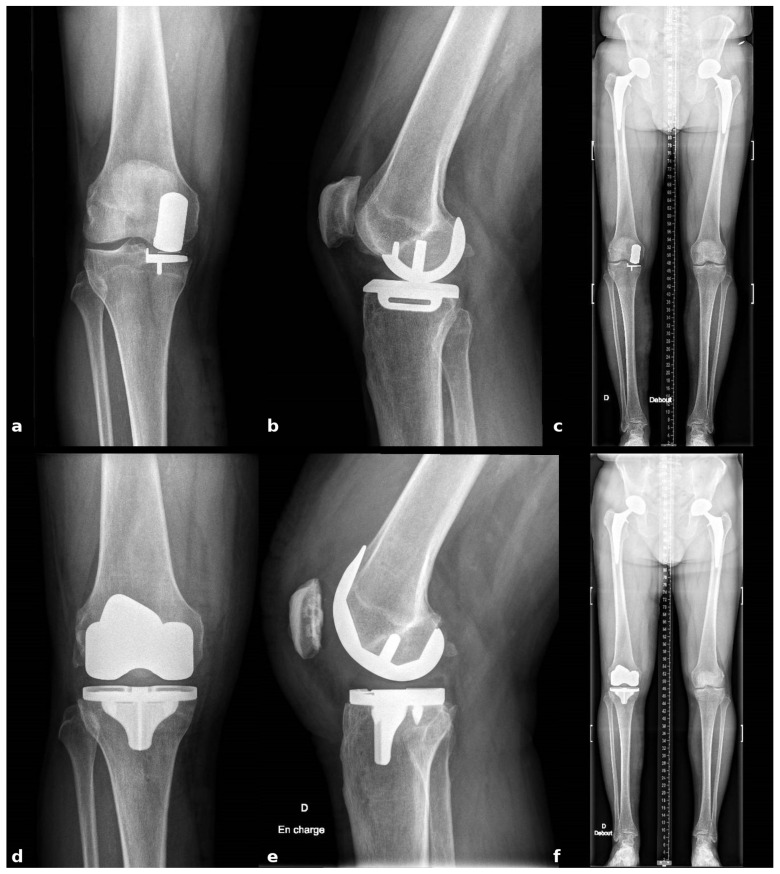
Radiographic comparison of preoperative and postoperative component positioning and alignment (**a**) Preop AP view; (**b**) Preop Lateral view; (**c**) Preop Long-leg alignment view; (**d**) Postop AP view; (**e**) Postop Lateral view; (**f**) Postop Long-leg alignment view.

Postoperatively, patients follow a standardized rehabilitation program that encourages immediate mobilization and full weight-bearing. Deep venous thrombosis prophylaxis and early quadriceps activation exercises are initiated on the day of surgery. Postoperative radiographs are obtained to confirm accurate component positioning and restoration of limb alignment (Figure 9).

## 3. Results

The described workflow enables stable registration despite metallic artifacts from the in situ UKA components and allows bone-sparing implant removal. Functional-alignment planning combined with robotic guidance achieves symmetric extension and flexion gaps without ligament or soft-tissue releases. Localized metaphyseal defects at the former tibial keel site are managed with impacted autologous cancellous graft harvested from femoral resections. Final implant placement matched the preoperative plan, and postoperative radiographs confirmed restoration of limb alignment and appropriate component positioning.

## 4. Discussion

Conversion of UKA to TKA remains technically demanding, especially when the aim is to use standard primary implants. These cases frequently share the complexity, risks, and perioperative management considerations of revision procedures due to pre-existing bone resections, retained implants, and altered joint mechanics, often related to malpositioning or wear. Even in the absence of infection, wear-induced osteolysis or loosening, stems and augments could be required [2,3,4].

Robotic assistance offers distinct advantages in this particular context. The combination of CT-based 3D planning, real-time ligament balance assessment, and precise bone resections supports an individualized and bone-preserving strategy. In contrast to conventional mechanical alignment, which may position cuts away from healthy bone and thereby necessitate stems or augments, functional alignment aims to restore native bone morphology and the surrounding soft-tissue envelope. This maximizes the available bone stock for fixation and supports the use of cementless implants while minimizing the need for additional augmentation [5,6,7,9].

Magruder et al. demonstrated that performing pre-explantation registration directly on the UKA components is technically feasible and allows for maintaining registration accuracy despite CT metal artifacts [8]. In our workflow, this concept is combined with a functional alignment strategy to preserve bone stock, maintain soft-tissue balance, and achieve stable fixation on healthy bone without the use of stems or augments. By acquiring landmarks along the sagittal central axis of the implants before removal, accurate mapping of both femoral and tibial anatomy can be achieved despite artifact interference. Successful execution of this technique requires proficiency with robotic workflows, and the learning curve for UKA-to-TKA conversion should be taken into account [6,10,11].

Management of limited bone defects with autologous cancellous bone grafting is incorporated to restore a stable platform for tibial baseplate fixation without stems or augments. This combination of robotic pre-explantation registration, functional alignment planning, and bone-preserving defect management offers a reproducible pathway for accurate reconstruction in selected UKA-to-TKA conversions [5,6,8,9].

Although this report focuses on the technical workflow, the combination of functional alignment and cementless fixation may translate into improved implant longevity and reduced need for future revision, particularly in younger or more active patients, and further comparative studies are warranted to determine whether this bone-sparing robotic approach influences long-term functional outcomes, implant survival, and revision rates compared with conventional conversion techniques.

While robotic-assisted UKA-to-TKA conversions have been described, few reports have outlined such a detailed, stepwise workflow combining these elements to allow the exclusive use of standard cementless components [5,6,7,8,10,11]. This protocol may offer a reliable and reproducible option for carefully selected patients.

This bone-preserving workflow is intended for patients with preserved metaphyseal bone stock (containing defects amenable to primary resections), competent collateral ligaments, and no active or suspected periprosthetic joint infection. Deformity should be correctable within our functional-alignment targets without the need for stems or augments. The technique is not recommended in the presence of extensive segmental bone loss, collateral ligament insufficiency, or persistent instability across the arc of motion, periprosthetic joint infection, or compromised bone quality, where revision-type constructs are preferred.

This technical note is limited by its descriptive nature and absence of long-term clinical outcome data. In addition, the technique requires proficiency with robotic workflows and may involve a learning curve for surgeons unfamiliar with these. Larger prospective studies are needed to validate reproducibility, assess functional outcomes, and determine implant survival compared with conventional conversion techniques.

## 5. Conclusions

This technical note outlines a reproducible, bone-preserving, robot-assisted workflow for the conversion of medial UKA to TKA using standard cementless condylar-stabilizing components, without the need for stems or augments. The procedure integrates 3D CT-based planning, pre-explantation registration on in situ implants, and dynamic soft-tissue assessment to enable functional alignment and secure fixation on healthy bone. Key targets include a hip–knee–ankle angle of 180° ± 5°, maintenance of the joint-line height within ±2 mm, and balanced gaps of 1–2 mm verified under standardized varus–valgus stress in terminal extension and at 90° of flexion. This approach is intended for carefully selected patients with preserved metaphyseal bone stock, competent collateral ligaments, and no active or suspected periprosthetic joint infection. Cases with extensive bone loss, ligament insufficiency, infection, or compromised bone quality are more appropriately managed with revision-type constructs. Given the learning curve, we suggest performing this technique in centers experienced with robotic TKA workflows and UKA-to-TKA conversion. As a descriptive technical report, outcomes are not presented here. A consecutive case series is underway to refine indications and evaluate safety, effectiveness, and survivorship.

## Data Availability

No datasets were generated or analyzed. De-identified operative images underlying the figures are available from the corresponding author upon reasonable request, subject to institutional and patient-privacy restrictions.

## Abbreviations

The following abbreviations are used in this manuscript:

UKA	Unicompartmental Knee Arthroplasty
TKA	Total Knee Arthroplasty
CS	Condylar-Stabilizing
